# Diagnostic and stewardship considerations in fungal lower urinary tract infections of dogs and cats

**DOI:** 10.1093/jvimsj/aalag284

**Published:** 2026-10-27

**Authors:** Cameron Prior, Jane E Sykes, Vanessa R Barrs, Riina Rautemaa-Richardson, Lena Klingspor, Oliver A Cornely, Tamara Gull, Fergus Allerton, Nelesh P Govender, J Scott Weese

**Affiliations:** Evidensia Södra Djursjukhuset Kungens Kurva, 141 75 Huddinge, Sweden; Department of Medicine & Epidemiology, Weill School of Veterinary Medicine, University of California, Davis, CA 95616, United States; Department of Veterinary Clinical Sciences, and Centre for Animal Health and Welfare, City University of Hong Kong, Kowloon Tong, Hong Kong SAR, China; Mycology Reference Centre Manchester, ECMM Centre of Excellence, and Department of Infectious Diseases, Manchester Academic Health Science Centre, Wythenshawe Hospital, Manchester University NHS Foundation Trust, Manchester M23 9LT, United Kingdom; Division of Evolution, Infection and Genomics, Faculty of Biology, Medicine and Health, University of Manchester, Manchester M23 9LT, United Kingdom; Division of Clinical Microbiology, Department of Laboratory Medicine, Karolinska University Hospital Huddinge, Karolinska Institutet, Stockholm, Sweden; Institute of Translational Research, Cologne Excellence Cluster on Cellular Stress Responses in Aging-Associated Diseases (CECAD), Faculty of Medicine, University of Cologne, Cologne 50923, Germany; Department I of Internal Medicine, Division of Infectious Diseases, Excellence Center for Medical Mycology (ECMM), Faculty of Medicine, University Hospital Cologne, Cologne 50923, Germany; German Centre for Infection Research (DZIF), Partner Site Bonn-Cologne, Cologne 38124, Germany; Faculty of Medicine and University Hospital Cologne, Clinical Trials Centre Cologne (ZKS Köln), University of Cologne, Cologne, Germany; Bacteriology/Mycology, Veterinary Medical Diagnostic Laboratory (VMDL), University of Missouri, 901 E. Campus Loop, Columbia, MO 65211, United States; Willows Veterinary Centre & Referral Service, Solihull B90 4NH, United Kingdom; Wits Mycology Division, Faculty of Health Sciences, University of the Witwatersrand, Johannesburg 2050, South Africa; Institute for Infection and Immunity, City-St George’s University of London, London NW3 2QG, United Kingdom; MRC Centre for Medical Mycology, Exeter EX4 4QD, United Kingdom; Division of Medical Microbiology, Falmouth Building, UCT Medical School, Anzio Road, Observatory, Cape Town, 7925, South Africa; Department of Pathobiology, Ontario Veterinary College, University of Guelph, Guelph, ON N1G 1R7, Canada

**Keywords:** microbiology, cystitis, renal/urinary tract

## Abstract

Fungal cystitis in dogs and cats presents a diagnostic and therapeutic challenge, with implications for antifungal stewardship in veterinary medicine. Unlike bacterial cystitis, diagnostic pathways for fungal infection are less well-defined, and antifungal therapy is often empirical. This article discusses key considerations for the diagnosis and management of fungal urinary tract infections, including the interpretation of urine cytology, the potential role and limitations of fungal culture, and the value of species-level identification and antifungal susceptibility testing. Clinical, laboratory, and pharmacokinetic factors that influence treatment decisions are highlighted, alongside the effect of confounding host and iatrogenic risk factors. The article also explores current gaps in evidence, variability in laboratory capabilities, and opportunities for strengthening antifungal stewardship in veterinary practice. Rather than providing prescriptive recommendations, this work aims to raise awareness, facilitate informed decision-making, and guide future research and consensus-building efforts to improve outcomes for animals while contributing to broader antifungal stewardship initiatives.

## Background and rationale

The term funguria refers to the detection of fungal organisms in urine by microscopic examination of urine sediment or culture^1^ and does not include the detection of fungal antigens in urine (such as urine *Blastomyces* antigen). In some cases, when environmental contamination can be excluded, fungi might be present in urine in the absence of clinical signs of urinary tract disease (subclinical funguria). Distinguishing this from infection is important, as subclinical funguria might not warrant antifungal treatment.^2^ When detection of fungal organisms is associated with compatible clinical signs and evidence of urinary tract inflammation, the condition is considered fungal cystitis.^1^

*Candida* spp. and closely related yeasts (former *Candida* species) are the most commonly isolated yeasts that cause fungal cystitis in dogs and cats, with *Candida albicans* and *Nakseomyces glabratus* (formerly *Candida glabrata*) most commonly isolated; however, other yeasts, and, less commonly, molds or dimorphic fungi might also be involved.^3–5^ Antifungal susceptibility varies among species: for example, *Pichia kudriavzevi* (formerly *Candida krusei*) and *Aspergillus* spp. have intrinsic resistance to fluconazole, while acquired azole resistance is reported in *Candida tropicalis* and *N. glabratus* isolated from dogs with urinary tract infections.^6^^,^^7^ These differences highlight the need for species identification and susceptibility testing.

Antifungal resistance is an increasing concern in both human and animal health.^8^^,^^9^ In human medicine, antifungal stewardship programs demonstrate that it is possible to reduce antifungal use without adversely affecting outcomes in both high- and low-resource settings.^8^^,^^10^^,^^11^ Central to these successes is the integration of antifungal stewardship principles with timely and accurate diagnostic testing, allowing treatment to be targeted, reviewed, and refined.

Comparable antifungal stewardship frameworks are largely absent in veterinary practice. Awareness of fungal disease among veterinarians is well-established for superficial mycoses such as dermatophytosis and *Malassezia* infections, but there is a relative lack of awareness for systemic fungal infections, including fungal cystitis.^12^ This could restrict the quality and quantity of clinical and laboratory data available for monitoring fungal disease prevalence and resistance trends in fungal urinary tract infections (FUTI) specifically.^13–15^

Fungal urinary tract infection in dogs and cats is uncommon and most often occurs in the presence of predisposing host or iatrogenic factors that disrupt normal urinary tract defense mechanisms.^3^^,^^4^ Reported risk factors include indwelling urinary catheters, recent or prolonged antibacterial therapy, hospitalization, and urological procedures, all of which could facilitate fungal colonization or introduction of opportunistic organisms.^5^ Systemic diseases such as diabetes mellitus, hyperadrenocorticism, chronic kidney disease, and treatment with immunosuppressive drugs might further increase susceptibility.^5^ Structural or functional abnormalities of the urinary tract, including urolithiasis, neoplasia, urinary obstruction, and incomplete bladder emptying can also promote persistence of fungal organisms within the urinary tract or alter the local environmental and protective mechanisms.^5^^,^^16^^,^^17^ From a stewardship perspective, identifying and addressing predisposing factors is a key component of management, particularly in animals with recurrent fungal infections. Correction of these factors will help reduce the risk of fungal colonization and recurrence.

Funguria does not necessarily indicate fungal disease. As with subclinical bacteriuria, subclinical funguria can be present and be clinically irrelevant; *Candida* spp. are the organisms most often implicated as incidental urinary colonizers in this contex.^4^^,^^18^

Clinicians should evaluate the animal’s clinical signs, underlying risk factors (such as immunosuppression, prolonged antibiotic use, or indwelling urinary catheters), the potential for concurrent disease (eg, bacterial infection, urolithiasis, neoplasia) and urinalysis findings before initiating antifungal therapy.

The scope of this article is to provide an overview of issues and considerations for the diagnosis and management of fungal cystitis in dogs and cats. The discussion primarily addresses infections caused by yeasts, particularly *Candida* species, an opportunistic yeast capable of colonizing mucosal surfaces without causing disease, which are also the most frequently reported fungal pathogens associated with fungal cystitis in dogs and cats.^5^

## Considerations for urine sediment cytology in the diagnosis of fungal cystitis

Cytologic examination of urine sediment is an easy and accessible initial diagnostic tool for identifying funguria. Direct visualization of yeasts, pseudohyphae, or hyphae should prompt fungal culture rather than immediate antifungal treatment, as fungi are ubiquitous in the environment and can be common contaminants of voided urine samples. Culture alone cannot reliably distinguish contamination from true infection, since a contaminant organism can also grow, but interpreting culture results alongside clinical signs, cytological findings, and, where needed, repeat cystocentesis sampling helps make this distinction. Urine collected by cystocentesis is therefore preferred over voided samples when funguria is suspected, since it avoids contamination from the distal urethra, genital tract, and skin. Regardless of collection method, documenting fungal cytomorphology (eg, budding yeasts, pseudohyphae, true septate hyphae) is important, as morphology can narrow the differential diagnosis. For example, rare pigmented fungal hyphae identified in a voided sample are more likely to represent contamination than true infection. Septate hyphae raise concern for *Aspergillus* spp. whereas pseudohyphae admixed with budding yeast are more typical of *Candida* spp. Pyuria could be noted concurrently but is not diagnostic of infection, as subclinical funguria can occur with pyuria present; pyuria is not part of the case definition and should not, on its own, be used as a trigger for antifungal treatment.

The presence of concurrent inflammatory cells might strengthen the clinical relevance of funguria, though this has not been systematically studied. Unlike subclinical bacteriuria, which can occur with minimal or no concurrent pyuria,^18^ in the authors’ experience, marked pyuria (eg, neutrophils and macrophages too numerous to count per high-power field) with intracellular or phagocytosed fungal organisms is more suggestive of clinically relevant infection rather than incidental colonization or contamination; however, this has not been formally validated. In the absence of fungal-specific pyuria thresholds, cytological findings should be interpreted together with clinical signs and, where possible, confirmed with quantitative fungal culture from a cystocentesis sample.

## Considerations for fungal culture in diagnosis of fungal cystitis in dogs and cats

Fungal culture of urine confirms funguria and allows species identification, which can help predict antifungal susceptibility. Fungi can also be detected during routine bacterial urine culture, so clinicians might encounter funguria without specifically requesting fungal culture; the same principles of clinical correlation apply in this situation.

*Candida* spp. account for most FUTI cases in dogs and cats typically grow on standard bacterial urine culture media within a routine turnaround time, meaning a dedicated fungal culture request is not always necessary.^5^ However, fungal culture media could improve recovery of non-*albicans Candida* species on a fresh, cystocentesis-collected sample particularly in cases of concomitant bacterial and fungal UTI, the fungal pathogen might be overlooked because bacteria grow more rapidly in routine culture media.

Use of fungal culture media is also important when fastidious or filamentous fungi are suspected; the presence of these fungi in urine usually reflects an underlying systemic (disseminated) infection. Detection of *Aspergillus* spp., *Rasamsonia argillacea*, and *Scedosporium apiospermum* is improved through use of specific fungal culture media and prolonged incubation. Clinicians should suspect these organisms in animals with hyphae evident on cytology, poor response to standard antifungal therapy, or predisposing immunosuppression, and should communicate with the microbiology laboratory to ensure appropriate culture conditions are requested. Viable yeasts particularly *Cryptococcus* (encapsulated yeast), dimorphic fungi such as *Blastomyces*, *Histoplasma*, and *Coccidioides,* are rarely identified in urine by cytology and culture and few reports of cystitis exist. When these fungi are suspected based on geographic location and clinical findings, urine or serum antigen testing (*Cryptococcus*, *Blastomyces, Histoplasma*) or serum antibody testing (*Coccidioides*) are often preferred for diagnosis, owing to their greater sensitivity, shorter turnaround time, and improved laboratory safety compared with attempting to culture a potentially hazardous dimorphic organism. Fungal culture nonetheless remains the diagnostic gold standard for definitively identifying the causative organism, and confirmation by culture, cytology, or histopathology should be pursued where feasible rather than relying on antigen or antibody results in isolation, particularly when results are only weakly positive; over-reliance on antigen or antibody testing without attempts at organism identification risks misdiagnosis.^19^ Given the potential for laboratory-acquired infection, suspicion for a systemic mycosis should always be communicated to the laboratory regardless of which diagnostic modality is used.

Where available, MALDI-TOF mass spectrometry offers rapid, reliable species-level identification of yeasts, but its performance for filamentous molds is less well established.^20^^,^^21^ Conventional biochemical methods, though more widely available, perform less reliably for uncommon species.^22^^,^^23^ Internal transcribed spacer or D1/D2 (large-subunit rRNA gene) sequencing could also be available at some laboratories, offering an alternative for isolates that remain unresolved by the above methods.

Negative growth on cultures despite the presence of fungal yeast-like cells, hyphae (or both) on cytology, can reflect fastidious organisms, suboptimal culture conditions, or sample handling issues, particularly if the original sample was voided rather than collected by cystocentesis. Repeat sampling by cystocentesis with a request for fungal culture is recommended. If uncertainty remains, pan-fungal PCR with sequencing might be considered, though sensitivity from urine is likely low and a negative result should not be relied upon to rule out infection; targeted antigen or antibody testing could also be indicated in these instances. As with culture, a positive PCR result might reflect contaminant fungal DNA and should be interpreted alongside cytomorphology and clinical context.

## Considerations for assessment of in vitro antifungal susceptibility testing

Antifungal susceptibility testing supports in dogs and cats care and antifungal stewardship by informing drug selection and avoiding unnecessary escalation of antifungal therapy. The absence of validated veterinary breakpoints for fungal pathogens complicates interpretation, but inferences can be made based on human breakpoints, minimum inhibitory concentrations (MICs) and available pharmacokinetic data for dogs and cats. While susceptibility testing of yeasts is commonly available at diagnostic laboratories, few laboratories offer susceptibility determination of filamentous fungi.

Indications for susceptibility testing include isolation of unusual pathogens such as non-*albicans Candida* species, molds or organisms with known variable antifungal resistance patterns (eg, *Candida glabrata*, or azole-resistant *Aspergillus fumigatus* harboring cyp51A mutations^13^^,^^14^), prior antifungal drug exposure, or poor clinical response. Susceptibility testing also provides essential epidemiological data to guide future empiric therapy.

Antifungal susceptibility testing is performed on planktonic (free-floating) organisms rather than biofilms. This distinction has clinical relevance because *Candida* spp. and other fungal uropathogens can form biofilms on mucosal surfaces and indwelling urinary catheters, and organisms within biofilms can be substantially more resistant to antifungal agents than the same organisms growing in their planktonic form. While the role of biofilms in infections and the impact on treatment is unknown, susceptibility testing results could overestimate the likelihood of treatment success in animals where biofilm is likely to be present, and removal of predisposing factors such as urinary catheters should be considered an integral part of management rather than an optional adjunct.^24^

## Considerations for drug selection and therapeutic drug monitoring of fungal cystitis

Antifungal drug selection for FUTI should prioritize agents that achieve therapeutic urinary concentrations, in addition to appropriate spectrum of activity, bioavailability, and tolerability, which vary considerably between antifungal classes.^25^^,^^26^

Fluconazole is the only azole excreted largely unchanged by the kidneys and achieves high urinary concentrations in urine,^27^^,^^28^ making it the preferred first-line agent for fungal cystitis confined to the lower urinary tract, provided the causative organism is susceptible. Fluconazole is available formulations for oral or intravenous administration. In contrast, itraconazole, voriconazole, and posaconazole undergo extensive hepatic metabolism and achieve low to negligible concentrations in urine,^27^^,^^29^ meaning there is no reasonable expectation of efficacy as first-line agents for FUTI confined to the lower urinary tract, despite their broader activity against molds and dimorphic fungi.

Clinicians must account for intrinsic resistance patterns when selecting therapy. As fluconazole is ineffective against molds,^30^ identification of a mold pathogen should prompt evaluation for renal parenchymal or upper tract involvement, since isolated lower urinary tract mold infection is uncommon; if confirmed, itraconazole or voriconazole (for *Aspergillus* spp., pending susceptibility results) might be appropriate given systemic tissue penetration, despite inadequate urinary concentrations, or amphotericin B could be preferred where urinary drug levels are a specific concern. Fluconazole is also ineffective against *Pichia kudriavzevii* due to intrinsic resistance, and an alternative agent is required when this organism is identified.^30^

Amphotericin B might be considered for animals with life-threatening systemic mycosis and concurrent FUTI, or where oral azole therapy is not tolerated or has failed, though formulation choice involves an important trade-off. The liposomal formulation is associated with lower risk of nephrotoxicosis^31^^,^^32^ and achieves moderate renal parenchymal concentrations, but achieves minimal urinary excretion and is therefore unlikely to be effective where clearance of lower urinary tract infection specifically is the goal.^27^ The deoxycholate formulation achieves higher urinary drug levels^28^ and so could be more appropriate where this is the primary concern, though this must be balanced against its greater nephrotoxic potential.^31^^,^^32^ Echinocandins (caspofungin, micafungin) are not renally excreted in active form and do not achieve adequate urinary concentrations, limiting their role in FUTI despite their utility in systemic candidiasis or aspergillosis. Terbinafine achieves limited urinary concentrations but is used in combination with azoles or amphotericin B for selected mold infections^33^; however, evidence supporting terbinafine as part of combination therapy remains limited.^34^

Prolonged antifungal therapy might be needed, increasing the importance of monitoring for toxicosis and drug interactions.^35^ Azoles inhibit mammalian CYP-450 enzymes, and there is the potential for interactions with ciclosporin, corticosteroids, digoxin, benzodiazepines, or macrolide antibiotics.

## Role of diagnostic laboratories in antifungal stewardship

Diagnostic laboratories play an important role in supporting appropriate antifungal use. Where species-level identification is available, this alone can guide treatment decisions. For example, identifying *Pichia kudriavzevi* signals innate fluconazole resistance without the need for susceptibility testing. When antifungal susceptibility testing is performed, results are reported as minimum inhibitory MICs, the lowest concentration of a drug that inhibits fungal growth in vitro.

To translate these values into clinical guidance (susceptible, intermediate, or resistant), interpretive breakpoints are required. These breakpoints define the MIC threshold above which treatment failure becomes more likely and are ideally derived from a combination of pharmacokinetic data, clinical outcome studies, and microbiological data for each species.

In veterinary medicine, validated species-specific breakpoints are absent. Where susceptibility interpretation is attempted, human breakpoints are typically extrapolated, which might not fully account for differences in drug pharmacokinetics between species. Clinicians should therefore interpret susceptibility results cautiously, and where possible seek guidance from a veterinary clinical microbiologist or infectious disease specialist. Cascade reporting, where laboratories report only the most appropriate first-line agents initially, reserving broader agents for confirmed resistance or treatment failure can further support rational antifungal prescribing. Encouraging open dialogue with diagnostic laboratories about available tests and their limitations is an important and underutilized component of antifungal stewardship in veterinary practice.

## Future directions

Despite growing awareness of fungal disease in companion animals, important gaps remain in how it is diagnosed and treated.

Better outcome data are needed. Existing data are retrospective and limited in size, making it difficult to identify reliable predictors of treatment failure or to determine which animals are most likely to benefit from early escalation of therapy. Well-designed prospective studies would provide the clinical outcome data needed to move current management recommendations onto a more robust evidence base.

Veterinary-specific pharmacokinetic studies are required to develop the validated susceptibility breakpoints that are needed to reliably interpret laboratory resistance testing results in dogs and cats. Molecular diagnostic tools are advancing rapidly in human mycology and are beginning to find their way into veterinary practice. Tests such as pan-fungal PCR with sequencing can identify organisms that are missed on routine culture and are particularly valuable in complex or treatment-refractory cases.

Ensuring that veterinary clinicians know when to use these tools and how to access them, including through referral to specialist diagnostic laboratories, will become increasingly important. Even in well-resourced human medicine settings, advanced diagnostics are often centralized and performed offsite, causing delays. Veterinary medicine faces the same challenges, and investment in accessible diagnostic infrastructure will be essential.^36^

Surveillance of antifungal resistance in animal populations is currently limited. Without systematic monitoring of which fungal species are circulating and how their susceptibility patterns are changing over time, it is difficult to know whether current treatment approaches remain appropriate or whether resistance is quietly emerging.

Finally, structured antifungal stewardship programs designed specifically for veterinary practice do not yet exist. Managing fungal disease well requires combining multiple diagnostic approaches culture, cytology, species identification, susceptibility testing, and clinical judgment rather than relying on any single test or empirical treatment. Developing, testing, and sharing stewardship frameworks that are practical for everyday veterinary use should be a shared goal for the profession.

## Off-label antimicrobial declaration

Authors declare no off-label use of antimicrobials.

## Institutional animal care and use committee or other approval declaration

Authors declare no institutional animal care and use committee or other approval was needed.

## Abbreviation

FUTI: fungal urinary tract infections
